# Correlation of Clinical and Histopathological Features of Salivary Pleomorphic Adenoma

**DOI:** 10.30476/dentjods.2022.96307.1933

**Published:** 2023-12-01

**Authors:** Soussan Irani, Arash Dehghan, Zohreh Kalvandi

**Affiliations:** 1 Dept. of Oral Pathology, Dental Faculty, Dental Research Centre, Hamadan University of Medical Sciences, Hamadan, Iran. Lecturer at Griffith University, Gold Coast, Australia; 2 Anatomical Pathologist, Hamadan University of Medical Sciences, Hamedan, Iran; 3 Dentist, Private Clinic, Hamedan, Iran

**Keywords:** Histology, Neoplasm, Pleomorphic adenoma, Salivary glands

## Abstract

**Statement of the Problem:**

Salivary gland tumors represent about 3% of the head and neck tumors. Pleomorphic adenoma (PA) is the most common benign salivary gland tumor.

**Purpose:**

This study was conducted to investigate and describe some clinical and histopathological aspects of salivary pleomorphic adenomas with special reference to the epithelial and mesenchymal components.

**Materials and Method:**

In this retrospective study, one hundred tissue samples diagnosed as PA were sourced from archival tissue blocks between 2009 and 2019 in this retrospective study. Some clinical and demographic features, including age, sex, tumor size, and tumor location were recorded. This study included only samples taken by excisional biopsy. Then, the samples were histologically classified into three subtypes according to the proportion of epithelial and stromal components. The demographic and clinicopathological variables were statistically analyzed using Chi-square test or Fisher’s exact test, considering a significance level of 5% (*p*< .05).

**Results:**

In the present study, most cases (61%) were found in females, representing a female–male ratio of 1.6:1. The peak incidence was seen in the 4th and 5th decades of life. In 87% of cases, PA occurred in major salivary glands. There was a significant difference between the age of the patient and squamous metaplasia (*p*= 0.036). There was also a significant difference between the size of tumor and the amount of myxoid stroma (*p*= 0.021). Extensive myxoid stroma was mostly seen in tumors larger than 3.37cm (*p*= 0.001). In addition, there was a statistically significant difference between capsular invasion and the development of squamous metaplasia (*p*= 0.001).

**Conclusion:**

In this study, there was a significant correlation between the gland type and capsular features and between the size of tumor and rate of squamous metaplasia. A detailed clinical and histopathological analysis of PAs may provide a better insight to the pathophysiology of the lesion, tumor cell differentiation, and prognostic factors.

## Introduction

Salivary gland tumors represent about 3% of the head and neck tumors [ 1 - 2 ]. Pleomorphic adenoma (PA) is the most common benign salivary gland tumor, making up 40%-70% of all salivary gland tumors [ 3 ]. PA is mostly found in females and the average age at presentation is about 43 years [ 4 ]. The vast majority of cases arise in major salivary glands, predominantly the parotid gland. The palate is the most common site of minor salivary gland affected by PA. Histologically, PA is a mixed tumor composed of epithelial and myoepithelial components arranged in different morphologic patterns surrounded by a fibrous capsule [ 3 ]. The tumor stroma may appear as myxoid, chondroid, osseous and hyalinized. Depending upon which component predominates, PA can be classified into (I) cellular (either epithelial or myoepithelial cell rich) type, (II) mixed or classic type, and (III) stromarich (myxoid) type. However, it has been suggested that this classification lacks any clinical significance [ 5 ]. The epithelial tissue appears as ducts, strands, tubules, or solid sheets; it is classified into ductal-like cells and neoplastic myoepithelial cells. Myoepithelial cells appear as plasmacytoid, clear and spindle cells, squamous, sebaceous, and adipose metaplasia. Keratin pearl formation is another phenomenon, which is associated with squamous metaplasia. The stroma can be found as myxoid, chondroid, chondromyxoid, osseous, hyalinized, and fibrous tissue [ 6 ]. Due to some factors such as histological variability, common features to other salivary gland neoplasms and the variations in epithelial and stroma components, the detailed knowledge of histological patterns of PA may contribute to an accurate diagnosis of this tumor [ 6 ]. In the current study, we proposed to describe some clinical and histopathological aspects of salivary pleomorphic adenomas with special reference to the epithelial and mesenchymal components and to compare with those reported in prior studies. 

## Materials and Method

This retrospective study was approved by the local Research Ethics Committee (Protocol #IR.UMSHA.REC. 1397.290). One hundred tissue samples diagnosed as PA were recruited from archival tissue blocks between 2009 and 2019. This study included only samples taken by excisional biopsy. An anatomical pathologist and an oral pathologist reviewed all histopathologic slides to confirm the diagnosis. As several slides were prepared for each sample, all slides were reviewed carefully and then histological classifications of subtypes were provided. Some clinical and demographic features, including age, sex, tumor size, and tumor location (type of involved gland) were recorded. Then, the samples were histologically classified into three subtypes according to the proportion of epithelial and stromal components including Subtype I or cellular type, Subtype II or classic subtype, and Subtype III or myxoid subtype [ 6 ]. Then, the data were analyzed by descriptive analysis using SPSS (Statistical Package for Social Sciences) program, version 20. In addition, the clinical and histopathological variables were statistically analyzed using Chi-square test or Fisher’s exact test, considering a significance level of 5% (*p*< .05). 

## Results

**Figure 1:**
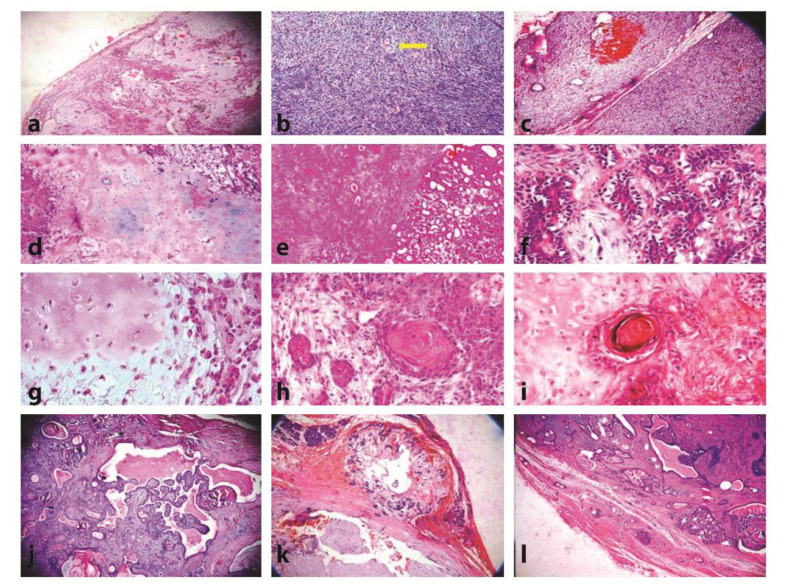
Histological patterns of PA, **a:** Classic type (100×), **b:** Cell-rich type. Remnant of a salivary duct is evident (100×), **c:** The myxoid component (100×), **d:** Areas of osseous and chondroid stroma(X100×), **e:** Extensive hyalinized area adjacent to the ductal pattern of tumor (100×), **f:** Clear myoepithelial cells (400×), **g:** Clusters of plasmacytoid-like cells (on the right) adjacent to chondroid and myxomatous stromal areas (X400), **h:** Squamous metaplasia (400×), **i:** Keratin pearl formation (400×), **j:** Cystic areas with papillary projections (100×), **k:** Pseudopedia adjacent to the main tumor capsule (100×), **l:** Capsular invasion(100×)

In the present study, 61 cases (61%) were found in females, representing a female–male ratio of 1.6:1. The tumors were distributed in a wide age range from 6 to 88 years, with a mean age of 38 years ± 16. In 6 cases, PA occurred in less than 16 years of age. The peak incidence was seen in the 4th and 5th decades of life. In 87% of cases, PA occurred in major salivary glands (77 cases in parotid). The hard palate was the most affected minor salivary gland (n=5), followed by the soft palate (n=3). The clinical and demographic data are presented in Table 1. Besides, all microscopic features are described in Table 2. Considering the histological subtype, Subtype I was observed in 32 cases, subtype II in 54 cases and subtype III in 14 cases. Clinical and histopathological characteristics of PAs have been compared based on affected salivary gland type. A detailed sumary are summarized in Table 3. In addition, there was a significant difference between the age of the patient and squamous metaplasia (*p*= 0.036). Squamous metaplasia was frequently found in patients older than 38 years. There was also a significant difference between the size of tumor and the amount of myxoid stroma (*p*= 0.021). Extensive myxoid stroma was mostly seen in tumors larger than 3.37 cm (*p*= 0.001). In addition, there was a statistically significant difference between capsular invasion and the development of squamous metaplasia (*p*= 0.001). Figure 1(A-I) shows the histopathological features of tumor.

**Table 1:** A summary of clinical parameters of 100 cases with pleomorphic adenoma

Clinical parameters	Pleomorphic adenomas (No.100)
Gender	
Female	61
Male	39
Female-to-Male ratio	1.6:1
Age(y)	
Mean age (range)	38(6-88)
≤38	57
>38	43
Diameter(cm)	
Mean (range)	3.37(1-9)
≤ 2	32
>2	68
Location	
Minor glands	13
Hard palate	5
Soft palate	3
Lip	2
Buccal	2
Oropharynx	1
Major glands	87
Parotid	77
Submandibular	10

**Table 2:** A summary of histopathological features of 100 cases with pleomorphic adenoma

Histological parameters	Number of cases
Subtype	
I	32
II	54
III	14
Lack of capsule/focal absence of the capsule	
Subtype I	5 (31.3%)
Subtype II	7 (43.8%)
Subtype III	4 (25 %)
Components and histologic findings	
Duct-like structure	95
Squamous metaplasia	39
Clear cells	28
Plasmacytoid-like cells	26
Keratin pearl	24
Spindle-shaped cells	18
Cystic formation	17
Adipose tissue	14
Myxoid stroma	73
Hyalinized stroma	46
Chondroid stroma	41
Osteoid stroma	36
Satellite Nodule	9
Pseudopedia	7
Mitosis	6

**Table 3:** Comparison of Demographic and Clinicopathological characteristics based on affected salivary gland type

Demographic and Clinicopathologicalsalivary salivary	Major salivary gland cases	Minor salivary gland cases	*p* Value
Gender			
Female	57	4	0.019*
Male	30	9	
Age(y)			
≤38	59	9	0.596
>38	28	4	
Size(cm)			
≤ 2	25	7	0.071
>2	62	6	
Capsule			
Lack /focal absence	9	7	0.001*
Complete	78	6	
Capsular invasion			
Yes	20	2	0.418
No	67	11	
Histopathologic subtype			
Cellular	30	2	0.205
Classic	44	10	
Stroma-rich	13	1	
Histologic components			
Squamous metaplasia	29	10	0.004*
Keratin pearl	18	6	0.054
Cystic formation	16	1	0.306
Hyalinized areas	35	11	0.003*
Plasmacytoma	24	2	0.286
Spindle cells	4	0	0.568
Clear cells	21	7	0.033*
Satellite nodules	8	1	0.669

* Results are statistically significant

## Discussion

PA is the most common benign salivary gland tumor. In addition, it is more common in females and commonly presents in the 5th and 6th decades of life [ 7 - 8 ]. In the present case series, the lesion was also, more common in females (61%) but 57% of the patients were aged 38 years and younger. Similar to other studies, in the current study, parotid gland was the most common affected salivary gland, followed by minor salivary glands [ 8 - 9 ]. The hallmark of PA is its histological diversity, which is composed of epithelial and stromal/mesenchymal component [ 10 ]. In accordance to a previously published paper, the present study showed myxoid stroma as the most frequent mesenchymal content, followed by hyaline, and chondroid stroma [ 9 ]. Other studies have indicated fibrous stroma as the most frequent stromal pattern, followed by myxoid stroma [ 11 - 12 ]. In the current study, lack of capsule or focal absence of the capsule was frequently manifested in Subtype II (43.8%). A previous meta-analysis has suggested that the stroma-rich tumors show a focal tumor capsule disruption and the formation of satellite nodules [ 13 ]. Similar to the study conducted by Wu *et al*. [ 6 ], in the present study, Subtype II (54%) was the most frequent histologic category, followed by Subtype I (32%). A previously published study found Subtype II (52%) as the most common histologic subtype, followed by Subtype III (28%) [ 14 ]. In a study carried out by Stennert *et al*. [ 15 ], Subtype III was reported as the most frequent subtype (51%) and Subtype II as the less frequent (14%) type. However, other studies have demonstrated Subtype I as the less common histologic subtype [ 14 , 16 ]. Squamous (epidermoid) cells and keratin pearl formation are also shown in PAs [ 17 ]. Squamous metaplasia occurs in about 20-25% of all PAs [ 18 ]. In the current study, squamous metaplasia was indicated in 39% of cases, mostly in major salivary gland tumors. In the present study, in accordance with previous research, the squamous metaplasia was associated with capsular invasion (*p*< 0.000) [ 19 ]. It has been suggested that trauma, ischemia, and tissue repair after infarction are the origin of squamous metaplasia [ 17 ]. A Previous investigation has shown that artery ligation results gradual dedifferentiation and hyperplasia of the acinar-intercalated duct system. Overtime, tonofilaments and desmosomes appear in the luminal and abluminal myoepithelial cells. Finally, keratinization of central cells happens [ 20 ]. The presence of squamous epithelium has been reported in a number of reactive or tumoral conditions such as necrotizing sialometaplasia, chronic sialoadenitis, Warthin's tumor, basal cell adenoma, and mucoepidermoid carcinoma [ 18 ]. It has been suggested that squamous metaplasia may increase the risk of development of squamous cell carcinoma [ 21 ]. In the present study, plasmacytoid-like cells were found in 26% of cases. A previously published study demonstrated that luminal cells are the origin of plasmacytoid-like cells [ 22 ]. A detailed study has indicated that plasmacytoid-like cells are in transition from one type of cell to another [ 5 ]. Other phenotype change is spindle shaped cells, which has been suggested as the possibility of epithelial-mesenchymal transition (EMT) phenomenon. This hypothesis has been raised from the previous investigations that considered the myoepithelial cells as the neoplastic cells. In addition, myxochondroid, osseous, or collagenous stromal variations have been suggested as their products. Therefore, EMT phenomenon may explain the dynamic transitions of tumor cells [ 23 ]. A previously published paper reported that in PA samples, plasmacytoid-like cells, and spindle cells express WT1, an important promoter of EMT [ 23 ]. Besides, in PA samples, E-cadherin expression was weak or absent in plasmacytoid-like cells and was negative in spindle cells [ 24 ]. These findings may suggest that plasmacytoid-like cells and spindle cells are capable of EMT. Thus, it could be concluded that plasmacytoid-like cell rich PAs and/or spindle cells rich PAs are more susceptible to malignant transformation. Therefore, it is advisable to be more careful in PA samples with higher frequency of plasmacytoid-like cells and spindle cells.

The age of patient, tumor size and location, incomplete excision or capsular violation can increase the risk of recurrence. We found a significant association between tumor size and myxomatous areas. Tumor size has been considered as a critical variable to predict of the malignant transformation in a salivary gland tumor [ 25 ]. On the other hand, myxoid areas are associated with more capsular disruption [ 23 ]. The capsular invasion and extension of tumor into the surrounding tissues through the capsule have been observed and called as satellite nodules. Pseudopodia and satellite nodules have been considered as the recognized causes of PA recurrence [ 25 ]. Interestingly, pseudopodia and satellite nodules have mostly been indicated in cellular type (Subtype I) PAs [ 15 ]. In other study, pseudopodia and satellite nodules were found in 46% of Subtype I cases and 18% of Subtype III cases, respectively [ 14 ]. In the present study, pseudopodia were detected in 7% of cases and satellite nodules were found in 9% of cases. Pseudopodia were found in 28% of Stennert *et al*.’s case series [ 15 ], in 40% of Zbären and Stauffer study [ 14 ], and in 20% of Grasso *et al*.’s samples [ 26 ]. Similar to other studies, we did not find any correlation between satellite nodules and pseudopodia and PA subtype [ 7 , 26 ].

## Conclusion

The present study demonstrated the diversity of histological characteristics of PAs. According to the results, duct-like structure was the most frequent histological finding. Besides, there was a significant correlation between the gland type and capsular features and between the size of tumor and the rate of squamous metaplasia. However, a larger sample size needs to be assessed in order to predict the possibility of malignant transformation. A detailed clinical and histopathological analysis of PAs may provide a better insight to the pathophysiology of the lesion, tumor cell differentiation, and prognostic factors.
